# THE FEASIBILITY OF USING COMMUNITY PHARMACISTS TO COUNSEL OLDER ADULTS ON FALL PREVENTION

**DOI:** 10.1093/geroni/igz038.1072

**Published:** 2019-11-08

**Authors:** Renae L Smith-Ray, Tanya Singh, Michael W Suwalski, Michael Taitel

**Affiliations:** 1 Walgreens Center for Health and Wellbeing Research, Deerfield, Illinois, United States; 2 Walgreen Co., Deerfield, Illinois, United States

## Abstract

Despite recognition as a serious public health problem, older adults falls increased 30% between 2007-2016. Numerous evidence-based fall prevention programs exist, but may have inadequate reach. Pharmacists are highly trained and accessible clinicians who have potential to counsel on fall prevention. This study describes the reach of a fall prevention outreach conducted by a large national pharmacy chain in partnership with local area agencies on aging (AAAs). On August 7, 2018, the pharmacy chain held an outreach during which older patients were incentivized to speak with pharmacists about their fall risk and prevention strategies. In Ohio, AAAs provided pharmacists additional support and availability of AAA fall prevention programs. A random sample of pharmacists was sent a follow-up survey to assess the program’s reach, except in Ohio where all pharmacists received the survey. Response rates were 41% (N=111) and 59% (N=160) in Ohio and non-Ohio states, respectively. We estimate that pharmacists discussed fall prevention with an additional 57,642 on 8/7/2018. The difference in older patients counseled on fall prevention on 8/7/2018 vs. a typical day was significantly greater (p=0.03) for Ohio pharmacists (µ=9.28) compared to non-Ohio pharmacists (µ=5.94). The majority of pharmacists in Ohio and non-Ohio states were moderately or extremely confident in their ability to discuss fall prevention with older patients (69.82% vs. 72.72%) and play an important role in fall prevention (59.75% vs. 54.54%). This study demonstrates the feasibility of utilizing community pharmacists, in partnership with AAAs, to reach large numbers of older adults to counsel on fall prevention.

